# Preparation of Stabilizer-Free Silver Nanoparticle-Coated Micropipettes as Surface-Enhanced Raman Scattering Substrate for Single Cell Detection

**DOI:** 10.1186/s11671-015-1122-x

**Published:** 2015-10-26

**Authors:** Yi-bin Tan, Jie-meng Zou, Ning Gu

**Affiliations:** Jiangsu Key Laboratory for Biomaterials and Devices, State Key Laboratory of Bioelectronics, School of Biological Sciences and Medical Engineering, Southeast University, 2 Sipailou, Nanjing, 210096 People’s Republic of China; Suzhou Key Lab of Biomedical Materials and Technology, Research Institute of Southeast University in Suzhou, 150 Ren Ai Road, Suzhou Industrial Park, Suzhou, 215123 People’s Republic of China

**Keywords:** Micropipette, Magnetron sputtering, Silver nanoparticles, SERS, Single cell detection

## Abstract

In this work, we established a convenient while reproduceable method for stabilizer-free silver nanoparticle (AgNP)-coated micropipettes by the combination of magnetron sputtering and surface coupling agent. The clear surfaces of the AgNPs are beneficial for absorbing biological or functional molecules on their surfaces. By optimizing the operating parameters, such as sputtering current and sputtering time, the tip of micropipettes coated with AgNPs exhibits excellent surface-enhanced Raman scattering (SERS) performance. Finally, the Raman spectra of a single A549 lung adenocarcinoma cell are successfully acquired by these advanced SERS-active micropipettes.

## Background

It is important to achieving detailed and accurate information of pathological process on cellular level in life science research. Generally, cell detection is based on indirect methods to collect certain components in a large amount of cells [1–3]. However, such statistically average results from a large amount of cells usually obscure the real mechanism of the cellular pathological process [4]. As a solution, detection of a single cell will help to comprehensively understand the individual difference and interaction [5, 6].

With the emerging of new technologies and instruments such as capillary electrophoresis, patch clamp, fluorescence microscope, and scanning tunneling microscope, the detection methods of a single cell have witnessed continuous development [7–10]. Raman spectroscopy, as a noninvasive optical detection method without ionizing radiation, has significant advantages like narrow spectrum peaks, interference immunity of water, hard quenching, and infrared light excitation compared with infrared spectroscopy and fluorescence spectroscopy, which is well suited to the research of biosystem in solution [11–14]. However, since the ratio between a Raman scattering event and the incident photons upon a molecule is extremely low (∼1 in 10 million), it is hard to obtain the Raman signals of a single cell. To conquer this problem, it is necessary to introduce nanostructured noble metals into the detection system. When the target moleculars absorbed on the surface of these metal nanostructures, the amplification of the Raman scattering signals would be improved to several orders. This phenomenon is also called surface-enhanced Raman scattering (SERS) [15–17]. Some SERS-based methods have been exploited to enhance the Raman signals of single cells [18–20].

Micropipettes made of pulling glass capillary can be used in microinjection of a single cell [21]. Also, it can be used to observe the electrophysiological activities of single cells in patch clamp experiments [22]. Using such micropipettes with a metal nanostructures coating on its surface can serve as a kind of SERS-active microprobe for single cell detection. Since there is no need to introduce external markers when using this kind microprobe to conduct Raman detection, the information from the cell itself and its chemical response to the environment can be obtained accurately and conveniently [23–25]. Vitol et al. [26] utilized the electrostatic adsorption of citrate-stabilized gold nanoparticles on the surface of micropipette tips to fabricate SERS probes for detecting cell nucleus and cytoplasm.

In this paper, a facile while robust method is developed to obtain stabilizer-free silver nanoparticle (AgNP)-coated micro-SERS probes based on magnetron sputtering process [27, 28]. Furthermore, we investigated the effects of preparation process on the SERS performances of the probe tips. Finally, SERS detection for single cells was conducted using the as-prepared micropipettes.

## Methods

### Preparation of SERS Micropipettes

Initial micropipettes were prepared through standard wall borosilicate tubing (1.0 mm outer diameter, 0.5 mm inner diameter, Sutter Instruments, Novato, CA). The instrument used to pull the tubing is P-97 Flaming/Brown micropipette puller (Sutter Instruments, San Rafael, CA). Micropipettes with controllable taper length and tip size can be obtained by adjusting the parameters like pulling strength and speed (PULL and VEL). The pulled micropipettes were cleaned by acetone and deionized water, respectively, and dried for further use. In order to improve the deposition rate of the metal covering layer and enhance its conjugation with the substrate, 3-aminopropyltriethoxysilanes (APTS) were adopted as the coupling agent according to the silver. The metal covering layer on the surface of micropipettes was produced by magnetron sputtering method through Q150TS Sample Preparation System (Quorum Technologies Limited Company, Kent, UK), which can operate on numerous micropipettes at the same time and realize mass production. The sputtering target material used in the system is silver (51 mm diameter, 1 mm thickness, 99.99 % purity, China New Metal Materials Technology, Beijing, China).

### Characterization Instruments

**Fig. 1:**
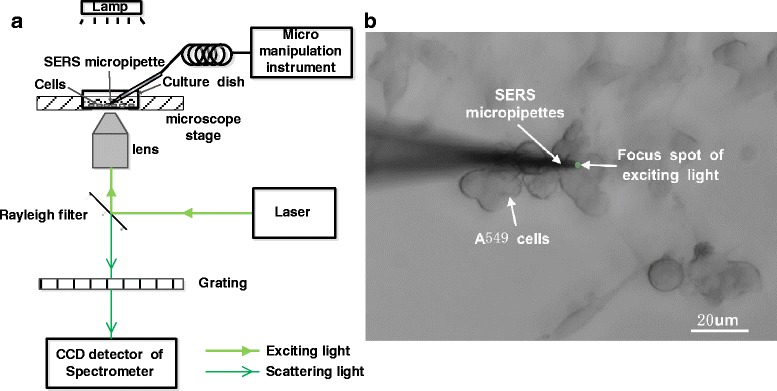
Raman detection system and single cell detection. **a** Diagrammatic drawing of the inverted microscope Raman detection system. **b** Photograph of the single cell Raman detection under optical microscopy with a 50× objective

The surface morphology of micropipettes with silver covering layer was imaged by a field emission scanning electron microscope (SEM, Zeiss Ultra Plus, Germany) with InLens capabilities. To overcome the limitation of the working distance of the objective lens, we have built an inverted micro-Raman detection system (Fig. 1a) to aim the micropipette stuck into the sample conveniently. The whole system consists of excitation light source (532 nm single longitudinal mode solid laser, AOTK, Xiamen, China), inverted microscope (DMI3000B, Leica, Germany), micromanipulator (MP-225, Sutter Instrument, San Rafael, CA), Raman spectrometer (inVia series, 1800 lines/mm grating, Renishaw, UK), and the spectral analysis software (Wire3.4, Renishaw, UK).

### Raman Spectra Acquisition of Single Cells Using SERS Micropipettes

Nile Bule A (NBA, China National Medicine Corporation Ltd., Shanghai, China) was used as Raman molecule for evaluating the SERS performances of different micropipettes. The concentration of NBA is 10^−5^ M and characteristic peak is 592 cm^−1^. The SERS micropipettes were immersed into the solution. The exciting source was focused on the sample through a ×50 telephoto objective lens to a spot size of approximately 2 μm. The Raman scattering was also collected by the same objective lens. The acquisition time for all spectra of micropipettes is 1 s, and the acquisition range is from 300 to 1000 cm^−1^.

Monolayer cultures of A549 cells, a human lung adenocarcinoma cell line, grew in the 35-mm culture dish with Dulbecco’s modified Eagle’s medium (DMEM, KeyGEN BioTECH Company, Nanjing, China). These cells were supplemented with 10 % fetal bovine serum (FBS, SiJiQing Biomaterial Company, Hangzhou, China), and the temperature was maintained at 37 °C in a humidified, 5 % carbon dioxide atmosphere. A SERS-active micropipette is stuck into the cell (Fig. 1b). The acquisition time for all spectra of cell is 10 s, and the acquisition range is from 400 to 2000 cm^−1^.

## Results and Discussion

### Raman Enhancement Influence of the Sputtering Parameters

In order to achieve great Raman enhancement, the plasmon resonance band of the SERS micropipettes should match the excitation wavelength [29]. It is directly related to the geometrical morphology of metal nanostructures on the substrate surface [30] and can be controlled by adjusting the sputtering parameters including sputtering current and sputtering time [31, 32].

**Fig. 2:**
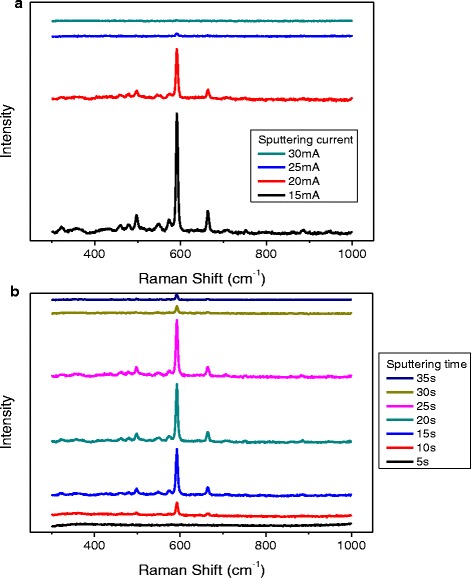
Raman spectra of micropipettes measured with NBA (10^−5^M). **a** Micropipettes prepared by different sputtering currents. **b** Micropipettes prepared using different sputtering times

Among these factors, sputtering current mainly affect the number of sputtering atoms and their mean kinetic energy. With the increased sputtering current, the mean kinetic energy of sputtering atoms will increase, which may promote the migration ability of sputtering atoms on the substrate surface. The combination between metal atoms and the substrate become more compact to form a smooth metal membrane, which is not preferable for the Raman enhancement. In this paper, we utilize different working currents and equal time to conduct the sputtering and choose the cylinder of the micropipettes to detect the SERS enhancement. By comparing the 592 cm^−1^ peak intensity of Nile Bule A (Fig. 2a), the obtaining result agrees well with the description above.

The influence of sputtering time on SERS enhancement was quite different from that of the sputtering current [27, 33, 34]. At the initial stage of sputtering, only a few of critical nuclei are formed on the base surface, and the size of metal particles is small. Here, the distance between particles is too large to enhance Raman signals by electromagnetic mechanism. When the distance between these metal particles reached the critical value to form electromagnetic enhancement, SERS signal appears and presents an increasing trend. When the metal nanostructure on the surface matched with the wavelength of the excitation light, the SERS enhancement achieves the peak value. By doing this, the surface of the substrate usually shows rough island-like membrane or network membrane structure. As the process of sputtering, continuous membrane started to form, and the roughness of substrate surface decreased, and the SERS enhancement would decrease gradually again (Fig. 2b).

### Raman Enhancement Influence of the Tip Geometry

**Fig. 3:**
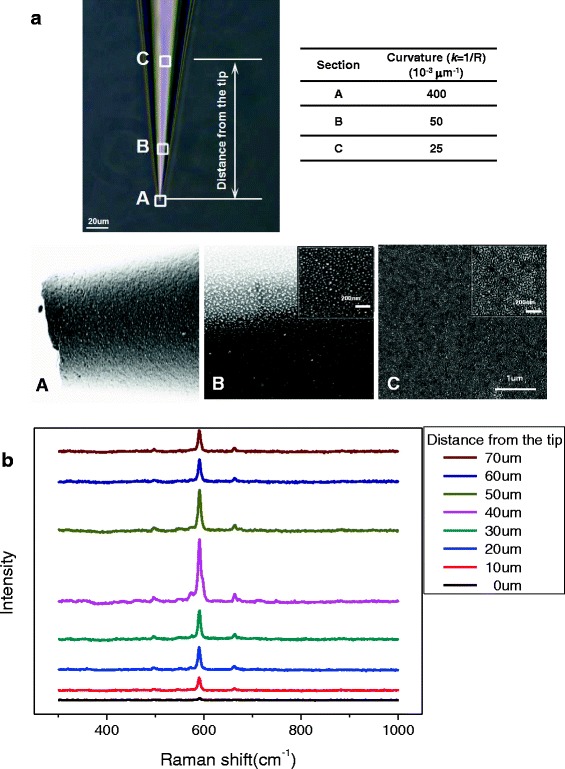
Characterization of the SERS micropipettes in different sections. **a** Photograph of optical microscopy and SEM. Three sections (*A*, *B*, *C*) of the micropipette with different curvatures are chosen for the observation of the film morphologies. The corresponding curvatures of sections A, B, and C are listed in the table. **b** Raman spectra of SERS micropipette with the detection location moved from the tip to the column end

For conical micropipette tips, in addition to the sputtering parameters, their own geometry also has great impact on the morphology of the metal covering layer. The tip of a SERS micropipette with a minimal diameter of about 2.5 μm is characterized under optical microscopy and SEM as shown in Fig. 3a. Three sections of the micropipette with different curvatures are chosen for the observation. Similar to the process of membrane forming with different sputtering times, critical nuclei, rough island-like membrane, and network membrane structure are found in the different sections. These phenomena show that the deposition of sputtering atoms at different sections of conical micropipette is determined by the curvature. It is more hardful for sputtering atoms attaching on the tip with large curvature than that on the column end. With the decrease of surface curvature, the formation of continuous film can be more quickly. SERS spectra of different sections also endorse this view. As the detection location moved from the tip to the column end, the SERS enhancement of micropipette presented an increasing-to-decreasing trend (Fig. 3b).

**Fig. 4:**
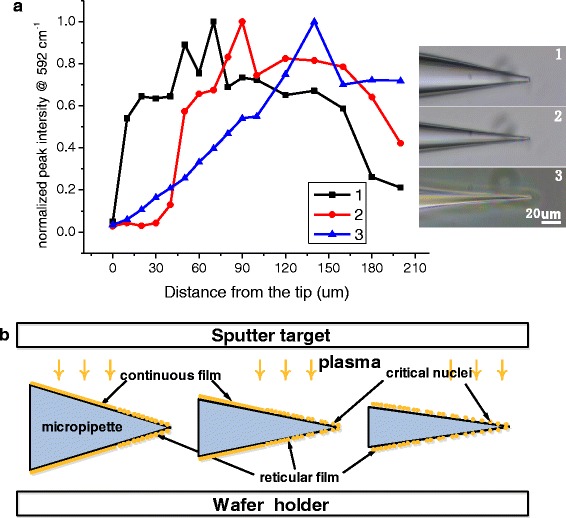
Characterization of the SERS micropipettes with different tip angles. **a** Comparison of the normalized peak intensity at 592 cm^−1^ from the tip to the column end on micropipettes. **b** The process schematic of formation of the metal membrane on micropipettes

Figure 4a shows the experimental results of similar SERS detection for a group of micropipettes with different taper angles but similar tip size. The result clearly indicated that, for single micropipette, the changing trend of characteristic 592 cm^−1^ peak intensity was the same as the result indicated in Fig. 3b. Seen from the comparison between tips with different angles, as the angle decreased, the changing speed of curvature from the tip to the column end was slowing down, and the location where the maximum 592 cm^−1^ characteristic peak intensity is increasingly far from the tip. The process schematic of formation of the metal membrane on micropipettes with different tip angles is given by Fig. 4b.

The above study and analysis were of vital importance in actual preparation of micropipettes. If micropipettes have better SERS enhancement, it would be quite helpful for obtaining weaker Raman signal at a single cell level. To achieve this goal, it is required to use different preparation parameters for micropipettes with different tip sizes. For this regard, we will fix the sputtering current and adjust the sputtering time to realize this goal.

### The Influence of Coupling Agent

**Fig. 5:**
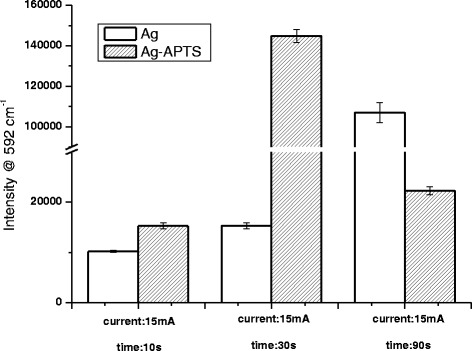
Characterization of SERS micropipettes prepared with or without coupling agent in different sputtering conditions

Without coupling treatment, the metal atoms generated by magnetron sputtering would attach to the substrate surface by physical absorption. As a result, it was not tight enough so that it is easy to be affected by the environment. When APTS is used as the coupling agent, monomolecular layer with regular shape is formed on the surface of micropipettes under waterless condition, thus being helpful for the attachment of sputtering atoms. To enhance the interaction between covering metal layer and substrate, a method of accelerating the deposition speed of sputtering atoms, reducing the sputtering time, and the wastage of target materials is used. Fig. 5 demonstrates the mean 592 cm^−1^ peak intensity of five groups of SERS micropipettes prepared with and without coupling agents. The result indicated that, with the presence of coupling agent, only a short sputtering time was required to obtain micropipettes with good SERS enhancement.

### Experimental Result of Single Cell Detection

**Fig. 6:**
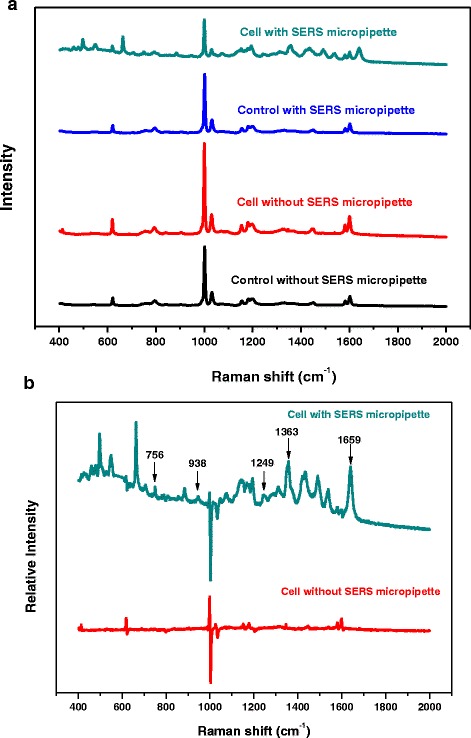
A549 cell detection with or without the SERS micropipettes. **a** Comparison of mean Raman spectra. **b** Comparison of the difference SERS spectra

We apply the prepared SERS micropipettes to conduct Raman spectrum detection on A549 cells. As shown in Fig. 6a, the cell culture environment such as culture dish and culture medium also have strong Raman signal, especially at 620,795,1001,1031,1154,1197,1448, and 1602 cm^−1^due to the biomolecule vibration, which would overlap the required information. Without SERS micropipettes, it is very difficult to obtain the weak Raman signal of A549 cells grew closely attaching to the bottom of the culture dish, even by confocal microscopy. Indeed, the Raman spectra of A549 cells without the presence of SERS micropipettes are nearly the same as the controlled spectra of the dish and medium (Fig. 6a). When SERS-active micropipettes are introduced, it can be clearly discovered that abundant characteristic peak signals attributed to the A549 cell including 756, 938, 1249, 1363, and 1659 cm^−1^ [35]. The above results clearly indicated that Raman signal can be enhanced at the tangent location of cell and micropipette. Through differential spectrum analysis and normalization processing, we can obtain richly spectroscopic information of the cells (Fig. 6b).

## Conclusions

In summary, we demonstrated a robust method of stabilizer-free AgNP-coated micro-SERS probes by magnetron sputtering and surface coupling modification. Using these SERS-active micro-substrate, we successfully obtain rich Raman signals of single cells in situ. This mass-production method owns a simple process, fast speed, and good repeatability. By adjusting the preparation parameters, we can optimize the SERS enhancement on the micropipettes and improve its detection sensibility. In future work, we will address the application of these SERS micropipettes for cell microinjection and electrophysiological detection, which is expected to realize a multi-parameter detection of single cells in situ.
